# On-X aortic valve replacement patients treated with low-dose warfarin and low-dose aspirin

**DOI:** 10.1093/ejcts/ezae117

**Published:** 2024-04-15

**Authors:** Aung Y Oo, Mahmoud Loubani, Marc W Gerdisch, Joseph Zacharias, Geoffrey M Tsang, Michael J Perchinsky, Robert Carl Hagberg, Mark Joseph, Mohanakrishnan Sathyamoorthy

**Affiliations:** Department of Cardiovascular Surgery, Bart’s Health NHS Trust, London, UK; Department of Cardiothoracic Surgery, Hull University Teaching Hospitals NHS Trust, Hull, UK; Department of Cardiothoracic Surgery, Franciscan Health, Indianapolis, IN, USA; Department of Cardiothoracic Surgery, Blackpool Teaching Hospitals Foundation Trust, Blackpool, UK; Department of Cardiac Surgery, Southampton General Hospital, Southampton, UK; Department of Surgery, Royal Jubilee Hospital, Victoria, BC, Canada; Department of Cardiac Surgery, Hartford Hospital, Hartford, CT, USA; Department of Surgery, Virginia Tech Carilion School of Medicine, Roanoke, VA, USA; Department of Internal Medicine, Burnett School of Medicine at TCU, Fort Worth, TX, USA

**Keywords:** On-X valve, Low-dose warfarin, Mechanical heart valve, Prosthetic valve, Anticoagulation, Aortic valve replacement

## Abstract

**OBJECTIVES:**

To assess if warfarin targeted to international normalized ratio (INR) 1.8 (range 1.5–2.0) is safe for all patients with an On-X aortic mechanical valve.

**METHODS:**

This prospective, observational registry follows patients receiving warfarin targeted at an INR of 1.8 (range 1.5–2.0) plus daily aspirin (75–100 mg) after On-X aortic valve replacement. The primary end point is a composite of thromboembolism, valve thrombosis and major bleeding. Secondary end points include the individual rates of thromboembolism, valve thrombosis and major bleeding, as well as the composite in subgroups of home or clinic-monitored INR and risk categorization for thromboembolism. The control was the patient group randomized to standard-dose warfarin (INR 2.0–3.0) plus daily aspirin 81 mg from the PROACT trial.

**RESULTS:**

A total of 510 patients were enroled at 23 centres in the UK, USA and Canada. Currently, the median follow-up duration is 3.4 years, and median achieved INR is 1.9. The primary composite end point rate in the low INR patients is 2.31% vs 5.39% (95% confidence interval 4.12–6.93%) per patient-year in the PROACT control group, constituting a 57% reduction. Results are consistent in subgroups of home or clinic-monitored, and high-risk patients, with reductions of 56%, 57% and 57%, respectively. Major and total bleeding are decreased by 85% and 73%, respectively, with similar rates of thromboembolic events. No valve thrombosis occurred.

**CONCLUSIONS:**

Interim results suggest that warfarin targeted at an INR of 1.8 (range 1.5–2.0) plus aspirin is safe and effective in patients with an On-X aortic mechanical valve with or without home INR monitoring.

## Graphical abstract

**Figure:**
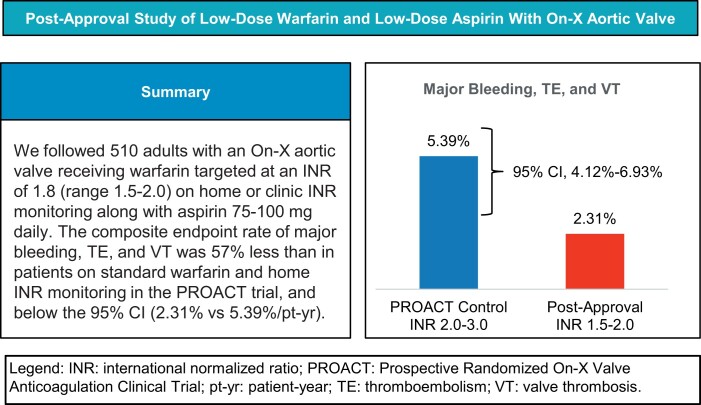

## INTRODUCTION

Patients with mechanical heart valves require lifelong anticoagulation with a vitamin K antagonist to minimize the risk of thromboembolism (TE) and valve thrombosis (VT) [1, 2]. Clinical trials of other antithrombotic agents, e.g. dabigatran, dual antiplatelet therapy with clopidogrel and aspirin, and apixaban, have not demonstrated efficacy for preventing thromboembolic events in patients with mechanical prosthetic heart valves [3–5]. With the exception of the On-X aortic mechanical valve (Artivion, Inc.), current guidelines recommend a target international normalized ratio (INR) of <2.5 for patients with an aortic bileaflet mechanical heart valve [2, 6]. In all patients with mechanical heart valves, the target INR should be individualized based on the type of mechanical valve and patient-related risk factors for TE [2, 6].

The On-X aortic valve has design features to reduce thrombogenicity, including material, flared inlet and leaflet angulation. It received CE Mark, Health Canada and US Food and Drug Administration (FDA) approval for use with anticoagulation targeted to an INR of 1.5–2.0, starting at least 3 months after valve implantation, based on results from the Prospective Randomized On-X Valve Anticoagulation Clinical Trial (PROACT) [4, 7, 8]. Daily aspirin (75–100 mg) and home INR monitoring are also recommended [9]; both were utilized in the PROACT trial. In the high-risk (HR) arm of the PROACT trial, patients with elevated risk factors for TE who had received an On-X aortic valve were randomized to low-dose (LD) warfarin (target INR 1.5–2.0) plus aspirin 81 mg daily or standard-dose (SD) warfarin (target INR 2.0–3.0) plus aspirin 81 mg daily, starting at least 3 months following On-X aortic valve implant. All patients receiving warfarin in the PROACT trial received a home INR monitor and were encouraged to use weekly home INR monitoring [4, 8]. Compared with patients treated with SD warfarin, those treated with LD warfarin had markedly lower rates of bleeding without a significant increase in thromboembolic events, resulting in an overall improved safety profile with LD warfarin.

As a condition of continued approval of the expanded labelling indication for the On-X aortic valve with INR 1.5–2.0, the FDA required a post-approval study (P000037 S030/PAS001) to ensure continued safety and efficacy. The protocol was developed by the sponsor and approved by the FDA. The study is designed to determine whether warfarin targeted to an INR of 1.5–2.0 is safe for all patients with an On-X aortic valve, regardless of TE risk level or monitoring method, and whether the results of the PROACT study are upheld during real-world use outside of a randomized control trial. To address these questions, this study (NCT02677974) evaluates rates of TE, VT and major bleeding (MB) in all-comer patients on LD warfarin (target INR 1.8, range 1.5–2.0) up to 5-year follow-up after implantation of an On-X valve. The primary objective is to compare adverse event (AE) rates in specified subgroups with those in the original PROACT trial. The secondary objective is to establish a better long-term safety profile of the On-X aortic valve. The prespecified study hypothesis predicts that the composite incidence rate of TE, VT and MB in the post-approval registry will be better than the 95% confidence bound of the rate in patients on SD warfarin (target INR 2.0–3.0) in the PROACT trial.

As of 16 May 2023, all 510 patients have completed or were scheduled to complete a minimum of 1 year of follow-up. This interim report describes these results through 16 May 2023. We believe the interim data regarding safety and efficacy are of value for surgeons or other clinicians who manage patients with an On-X aortic valve or who counsel patients considering an On-X aortic valve. The final results will be reported when all patients have completed 5 years of follow-up, anticipated in May 2027.

## PATIENTS AND METHODS

### Ethical statement

The study was conducted under the rules of the local country or the Declaration of Helsinki, whichever provides more patient protection. The Institutional Review Board or Ethics Committee at each site approved the study (Supplementary Material, Table S1). All enrolled patients provided written informed consent.

### Study design

This Post-Approval Registry is a prospective, multicentre, observational single-arm study designed to assess rates of AEs in patients receiving LD warfarin (target INR 1.8, range 1.5–2.0) during a 5-year period following On-X aortic valve implantation, after a minimum of 3 months of SD warfarin (target INR 2.5, range 2.0–3.0) postimplant. The study enrolled patients regardless of monitoring method or TE risk factors. Patients were to be recruited from 15 to 35 centres with appropriate expertise in Europe, the UK, Canada and the USA, starting in November 2015. Recruitment continued until the enrolment target was met in 2022. Each patient will be followed for 5 years.

### Thromboembolic risk classification

Patients with 1 or more of the following are classified as HR for TE:

- chronic atrial fibrillation
- left ventricular ejection fraction <30%
- enlarged left atrium >50 mm diameter
- spontaneous echo contrast in the left atrium
- neurological events
- hypercoagulability
- left or right ventricular aneurysm
- lack of platelet response to aspirin or clopidogrel
- oestrogen replacement therapy in female patients
- or vascular pathology (history of significant peripheral, carotid or coronary disease)

Patients without any of these risk factors are classified as low-risk (LR) for TE.

### Inclusion and exclusion criteria

Patients eligible for the study include:

- adults aged 18 years or older who have ‘only’ an On-X aortic prosthetic heart valve (ONXA, ONXAE, ONXAC, ONXACE, ONXAN and ONXANE) implant with or without concomitant procedures (Supplementary Material, Table S2)
- with a minimum life expectancy of 5 years
- whose operation occurred within 12 months before recruitment
- who agreed to participate

Patients excluded from the study are those:

- with any other type of prosthetic valve implant, either isolated or in combination with another valve(s), or any On-X mitral valve
- with history of arterial thromboembolic event(s) before valve surgery or recruitment or who develop VT after valve implant and before recruitment
- whose surgery predates enrolment by over 1 year
- who died prior to discharge or recruitment

To minimize bias, all patients meeting inclusion/exclusion criteria are offered enrolment.

### Intervention

The study observes patients receiving LD warfarin (target INR 1.8, range 1.5–2.0) following On-X aortic valve implant after a minimum of 3 months of SD anticoagulation. All patients with no contraindication to aspirin are prescribed daily aspirin (75–100 mg). Any patient experiencing a thrombotic event during the study will be returned to SD anticoagulation. Patients experiencing a bleeding event may or may not be continued on LD warfarin, depending upon the discretion of the clinician and principal investigator. All patients will be followed and will remain in the registry for analysis based on intent-to-treat. At 3 months postoperatively or at the time of enrolment if later than 3 months postimplant, patients decide, with their following and investigating physician(s), on the method of INR monitoring, i.e. at home or in a clinic. The patient and physician make all arrangements for home-monitoring including insurance coverage if applicable. For study purposes, a clinic is defined as a physician’s office, hospital laboratory or anticoagulation clinic.

### Control

The control is a historical randomized control group from the PROACT trial, i.e. those receiving SD warfarin (target INR 2.0–3.0) plus aspirin 81 mg daily following isolated On-X aortic valve replacement surgery, with or without concomitant procedures. The control group AE rates were predefined from data available when the post-approval study was designed. Data for the LR control group were obtained from the 2014 annual report of the PROACT trial (G050208); data for the HR control group were obtained from P000037/S030, a supplement to FDA’s premarket approval of On-X valve. The ‘composite’ control group combined data from high- and low-risk groups, and rates were calculated using a weighted average. These historical control rates defined in 2015 are listed in Supplementary Material, Table S3 and incorporated into Tables 2–4. These data differ slightly from the PROACT 2014 and 2018 publications due to the timeframe [4, 8].

**Table 2:** Post-approval study event rates versus PROACT control, all patients^a^

	LOR, %/pt-yr (no. of events)			
	Post-approval study, all, INR 1.5–2.0	PROACT composite control, INR 2.0–3.0	PROACT composite control, 95% CI	PAS versus control, IRR
Number of subjects	510	292		
Patient-years^b^	1562.9	1131.2		
Primary end point				
MB, TE, VT	2.31% (36)	5.39%	4.12–6.93%	0.43
Secondary and other end points				
MB	0.58% (9)	3.80%	2.75–5.12%	0.15
TE^c^	1.73% (27)	1.41%	0.81–2.30%	1.23
VT	0.00% (0)	0.18%	0.02–0.64%	0.00
All bleeding	1.92% (30)	7.07%	5.61–8.80%	0.27
TE and VT	1.73% (27)			
Minor bleeding	1.34% (21)			
Haemorrhagic stroke	0.13% (2)			
Ischaemic stroke	1.09% (17)			
Non-thrombotic ischaemic stroke	0.13% (2)			
TIA	0.58% (9)			
Peripheral TE	0.06% (1)			
Sudden death	0.13% (2)			
Valve-related mortality	0.00% (0)			
Total mortality	0.90% (14)			

a LORs are calculated based on late patient-years; end points occurring prior to low INR initiation are excluded.

b Patient-years in late follow-up, i.e. once LD anticoagulation initiated.

c Ischaemic stroke, TIA, plus peripheral TE.

CI: confidence interval; INR: international normalized ratio; IRR: incidence rate ratio; LOR: linearized occurrence rate; MB: major bleeding; PAS: post-approval study; PROACT: Prospective Randomized On-X Valve Anticoagulation Clinical Trial; pt-yr(s): patient-year(s); TE: thromboembolism; TIA: transient ischaemic attack; VT: valve thrombosis.

**Table 3:** Post-approval study event rates versus PROACT control by INR method^a^

	Linearized occurrence rate, %/pt-yr (number of events)			PROACT composite control, 95% CI
	Post-approval study, home INR, INR 1.5–2.0	Post-approval, study, clinic INR, INR 1.5–2.0	PROACT composite control, INR 2.0–3.0	
Number of subjects	70	440	292	
Patient-years^b^	209.7	1353.2	1131.2	
Primary end point				
MB, TE, VT	2.38% (5)	2.29% (31)	5.39%	4.12–6.93%
Secondary and other end points				
MB	0.00% (0)	0.67% (9)	3.80%	2.75–5.12%
TE^c^	2.38% (5)	1.62% (22)	1.41%	0.81–2.30%
VT	0.00% (0)	0.00% (0)	0.18%	0.02–0.64%
All bleeding	1.91% (4)	1.92% (26)	7.07%	5.61–8.80%
TE and VT	2.38% (5)	1.62% (22)		
Minor bleeding	1.91% (4)	1.26% (17)		
Haemorrhagic stroke	0.00% (0)	0.15% (2)		
Ischaemic stroke	2.38% (5)	0.89% (12)		
Non-thrombotic ischaemic stroke	0.00% (0)	0.15% (2)		
TIA	0.00% (0)	0.67% (9)		
Peripheral TE	0.00% (0)	0.07% (1)		
Sudden death	0.00% (0)	0.15% (2)		
Valve-related mortality	0.00% (0)	0.00% (0)		
Total mortality	0.48% (1)	0.96% (13)		

a LORs are calculated based on late patient-years; end points occurring prior to low INR initiation date are excluded.

b Patient-years in late follow-up, i.e. once LD anticoagulation initiated.

c Ischaemic stroke, TIA, plus peripheral TE.

CI: confidence interval; INR: international normalized ratio; LOR: linearized occurrence rate; PROACT: Prospective Randomized On-X Valve Anticoagulation Clinical Trial; pt-yr(s): patient-year(s); MB: major bleeding; TE: thromboembolism; TIA: transient ischaemic attack; VT: valve thrombosis.

**Table 4:** Post-approval study event rates versus PROACT control by TE risk category^a^

	Linearized occurrence rate, %/pt-yr (number of events)					
	Post-approval, high risk,^d^ INR 1.5–2.0	PROACT, high risk control, INR 2.0–3.0	PROACT, high risk, control, 95% CI	Post-approval, low risk, INR 1.5–2.0	PROACT, low risk, control, INR 2.0–3.0	PROACT, low risk, control, 95% CI
Number of subjects	128	190	190	382	102	102
Patient-years^b^	392.7	878.6	878.6	1170.2	252.6	252.6
Primary end point						
MB, TE, VT	2.55% (10)	5.80%	4.32–7.63%	2.22% (26)	3.96%	1.90–7.28%
Secondary and other end points						
MB	0.25% (1)	3.87%	2.68–5.41%	0.68% (8)	3.56%	1.63–6.76%
TE^c^	2.29% (9)	1.70%	0.96–2.82%	1.54% (18)	0.40%	0.01–2.21%
VT	0.00% (0)	0.23%	0.03–0.82%	0.00% (0)	0.00%	0.00–1.46%
All bleeding	1.27% (5)	7.85%	6.11–9.94%	2.14% (25)	4.53%	2.17–7.79%
TE and VT	2.29% (9)			1.54% (18)		
Minor bleeding	1.02% (4)			1.45% (17)		
Haemorrhagic stroke	0.00% (0)			0.17% (2)		
Ischaemic stroke	1.78% (7)			0.85% (10)		
Non-thrombotic ischaemic stroke	0.25% (1)			0.09% (1)		
TIA	0.51% (2)			0.60% (7)		
Peripheral TE	0.00% (0)			0.09% (1)		
Sudden death	0.25% (1)			0.09% (1)		
Valve-related mortality	0.00% (0)			0.00% (0)		
Total mortality	1.27% (5)			0.77% (9)		

a LORs are calculated based on late patient-years; end points occurring prior to low INR initiation date are excluded.

b Patient-years in late follow-up, i.e. once LD anticoagulation initiated.

c Ischaemic stroke, TIA, plus peripheral TE.

d See ‘Study Design’ Section for criteria for high-risk classification.

CI: confidence interval; INR: international normalized ratio; LOR: linearized occurrence rate; PROACT: Prospective Randomized On-X Valve Anticoagulation Clinical Trial; pt-yr(s): patient-year(s); MB: major bleeding; TE: thromboembolism; TIA: transient ischaemic attack; VT: valve thrombosis.

### End points

Study end point definitions are listed in Supplementary Material, Table S4 [10]. The primary end point is the composite rate of TE, VT and MB. The primary end point is reported overall and in 4 subgroups: INR monitored at home or in clinic, and high or low TE risk.

Secondary study end points include:

- individual components of the primary end point and TE plus VT
  - the primary composite end points TE, VT and MB in 4 subsets: HR home or clinic monitored and LR home or clinic monitored

Because these subsets were not randomized, statistical comparisons between subsets will not be performed.

Secondary events include: (i) death, reoperation or explant associated with a primary end point event, (ii) sudden death, (iii) minor bleeding requiring medical care and (iv) the number and proportion of subjects with INR readings <1.5 or >3.0.

Additional (tertiary) end points include the following clinically relevant events, which are reported overall, stratified by INR monitoring method and stratified by TE risk status: (i) all bleeding (major and minor), (ii) minor bleeding, (iii) haemorrhagic stroke, (iv) ischaemic stroke, (v) transient ischaemic attack (TIA) and (vi) peripheral TE. Reoperation, explant and death associated with ‘valve-related’ TE or VT, and bleeding are tertiary end points.

### Data and follow-up

Patients must agree to the proposed INR therapy, to visit their following physician 6 months after surgery and annually for 5 years, and to follow-up contact by the registry. They must agree that the site may contact their following physician to obtain data.

Data are collected prospectively for each patient for a 5-year follow-up period, with the exception of information from the 6-month postoperative visit, which may be obtained either prospectively or retrospectively for patients enrolled up to 12 months postoperatively. The study centre will contact each patient at 6 months postoperatively if enrolled, and then annually by telephone, text messaging, e-mail survey, or other accepted means of electronic communication. At each postoperative visit or electronic interaction, any valve-related complications will be documented, and the patient’s anticoagulant status will be reviewed including current target INR range and most recent INR value. If a patient experiences a primary end point event(s), their clinical records will be obtained for adjudication and classification by the committee.

### Statistical analyses

Sample size calculation assumed a one-sided test comparing the composite outcome to the reference value, a 5% significance level, at least 80% power, anticipated follow-up of 5 years per subject, 20% loss to follow-up over 5 years and >800 patient-years (pt-yrs) of follow-up in the HR group. The composite outcome incidence was estimated via Poisson regression. The expected overall composite rate of 4.57% per pt-yr was derived from the HR treatment (LD warfarin) arm of the PROACT trial. The reference value of 6.93% per pt-yr was based on the upper 95% confidence bound of the rate in the PROACT composite control group (HR and LR patients on SD warfarin) (Supplementary Material, Table S3). The sample size calculation yielded an enrolment target of 510 subjects with ∼816 pt-yrs in the HR group, if at least 40% of patients enrolled were HR.

Analyses used all available data and were performed using SAS software [11], version 9.4. Every effort was made to obtain complete data for all patients. Outcomes were analysed overall and by subgroups based on the INR method and TE risk category to permit accurate comparison with the PROACT trial, which was stratified by TE risk status and in which all patients were on home INR monitoring.

Early events, reported as simple percentages, were those occurring before initiation of LD warfarin; if that date was unknown, early events were those occurring up to 90 days post-implant.

Late events are those occurring after initiation of LD warfarin. Late events are reported as linearized occurrence rates and by Kaplan–Meier life tables. Linear occurrence rates for AEs were calculated based on number of events divided by the accumulated pt-yrs. Patients who withdrew were included up until the point of their withdrawal for the linearized occurrence rate calculations. Incidence rates for the composite and its components were analysed via Poisson regression. The Kaplan–Meier curve for freedom from a composite end point event was calculated from the initiation of LD warfarin until first end point event, and patients who had not experienced a primary end point event as of the date of last contact were censored at this date.

## RESULTS

The study enrolled 525 subjects at 23 centres in the UK, USA and Canada between November 2015 and January 2022. Enrolment was slower than initially anticipated, and was impacted by COVID-19 restrictions affecting medical centres worldwide. Fifteen subjects were deemed ineligible post-enrolment and subsequently exited due to: (i) 13 were implanted with an excluded On-X valve model (AAP, or ascending aortic prosthesis), (ii) 1 had a prior thrombotic event and (iii) 1 received aortic and mitral prosthetic valves. The remaining 510 subjects were included in the analysis. There were 128 (25.1%) at HR for TE and 382 (74.9%) at LR for TE, with INR home-monitored in 70 (13.7%) and clinic-monitored in 440 (86.2%).

The interim report includes data through 16 May 2023, which is when all patients had reached their 1-year follow-up visit. Median follow-up duration after initiation of LD anticoagulation is 3.35 years, with 1562.9 pt-yrs accumulated. Median achieved INR is 1.9. Overall follow-up is ∼90% complete for those subjects who were not discontinued for any reason. Dates of surgery spanned November 2014 through November 2021.

**Figure 1:**
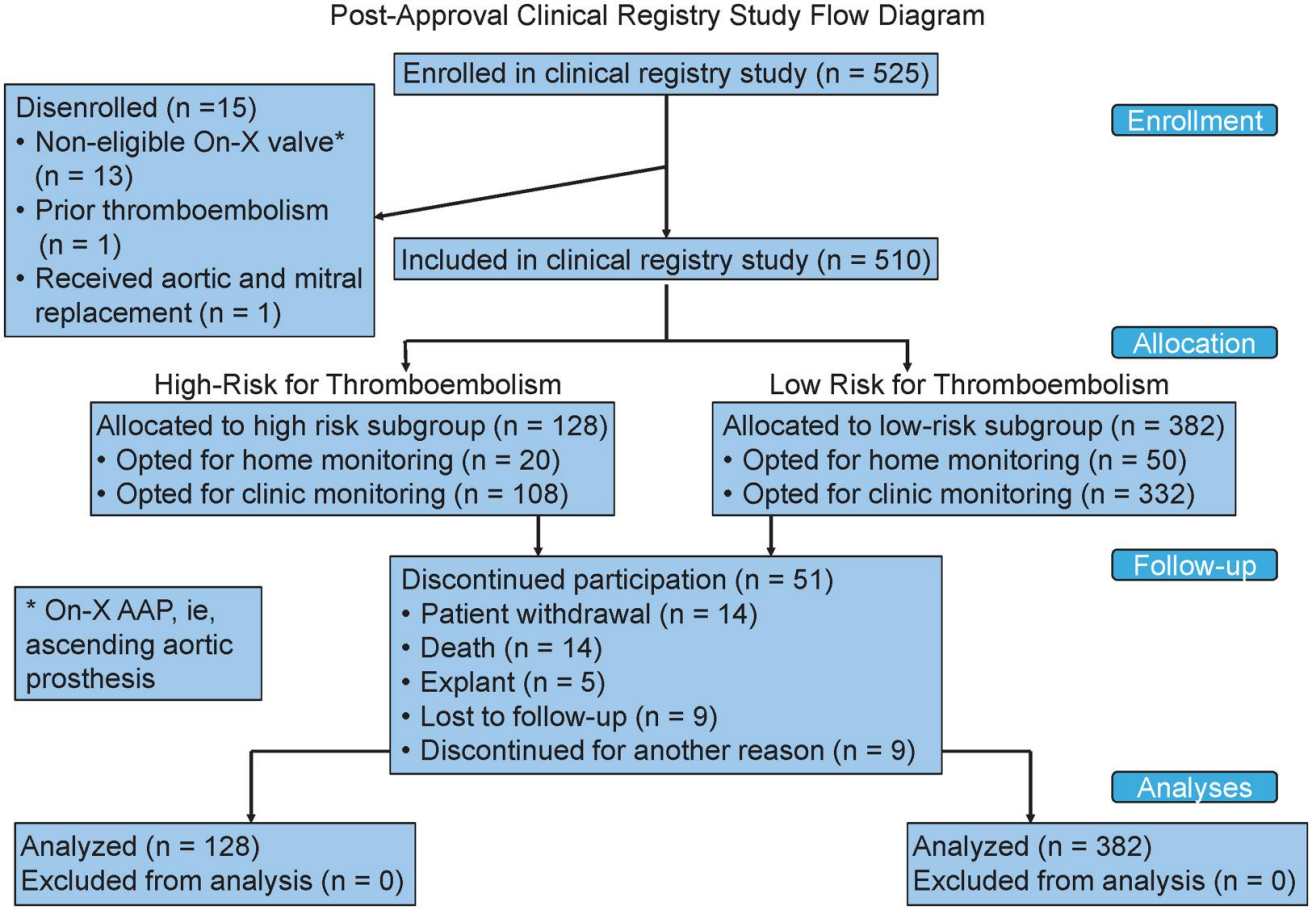
Study flow diagram. Flow of patients through the study is depicted, from enrolment through analysis.

Of 510 subjects, 51 discontinued participation during follow-up: 14 died, 14 were withdrawn due to investigator or subject decision, 9 were lost to follow-up, 5 experienced an explant and 9 discontinued for another reason (Fig. 1). The reasons for the 14 withdrawals by patient or investigator were not recorded. The reasons for the 9 patients discontinued for another reason were as follows: 7 patients were followed for 5 years but the 5-year follow-up visit was never completed or was completed after the patient purportedly finished the study, 1 patient discontinued follow-up at the study site and 1 patient enrolled in another study. For a complete description of the patients who discontinued from the study, please see Supplementary Material, Table S5 and Supplementary Material, Fig. S1.

### Baseline patient characteristics

Table 1 displays baseline demographic and clinical data. Mean patient age was 52.7 ± 11.1 years, with 72.9% male. Preoperatively, mean LVEF was 56.5 ± 10.4%, and 94.5% were in sinus rhythm.

**Table 1:** Baseline characteristics

Characteristic	All	Subjects assessed
Age		510
Mean ± SD (years)	52.7 ± 11.08	
Median (min, max) (years)	55.0 (20.0, 75.0)	
Sex, *n* (%)		510
Males	372 (72.9)	
Females	138 (27.1)	
NYHA class, *n* (%)		351
Class I	101 (28.8)	
Class II	177 (50.4)	
Class III	66 (18.8)	
Class IV	7 (2.0)	
Ejection fraction		460
Mean ± SD, %	56.5 ± 10.36	
Median (min, max), %	60.0 (6.0, 90.0)	
TE risk group, *n* (%)		510
Low	382 (74.9)	
High	128 (25.1)	
Cardiac rhythm, *n* (%)		510
Sinus	482 (94.5)	
Atrial fibrillation	9 (1.8)	
Paced	3 (0.6)	
Other	16 (3.1)	
Lesion, *n* (%)		510
Stenosis	272 (53.3)	
Regurgitation	127 (24.9)	
Mixed	103 (20.2)	
Other	8 (1.6)	
Prior cardiac surgery, *n* (%)		509
No	438 (86.1)	
Yes	71 (13.9)	

max: maximum; min: minimum; NYHA: New York Heart Association; SD: standard deviation; TE: thromboembolism.

### Early events

Early AEs, which occurred in 57 (11.2%) patients, are those occurring prior to lower INR implementation. Bleeding events occurred in 22 (4.3%), with 6 (1.2%) MBs, 3 (0.6%) minor bleeds, 11 (2.2%) postoperative bleeds and 2 (0.4%) traumatic bleeds (postoperative and traumatic bleeds not classified as major or minor). Non-cardiac events occurred in 27 (5.3%) patients. Other cardiac events occurred in 14 (2.8%) patients. An early TE, VT or death would have excluded a patient from the study.

### Primary study end point

#### Overall

**Figure 2:**
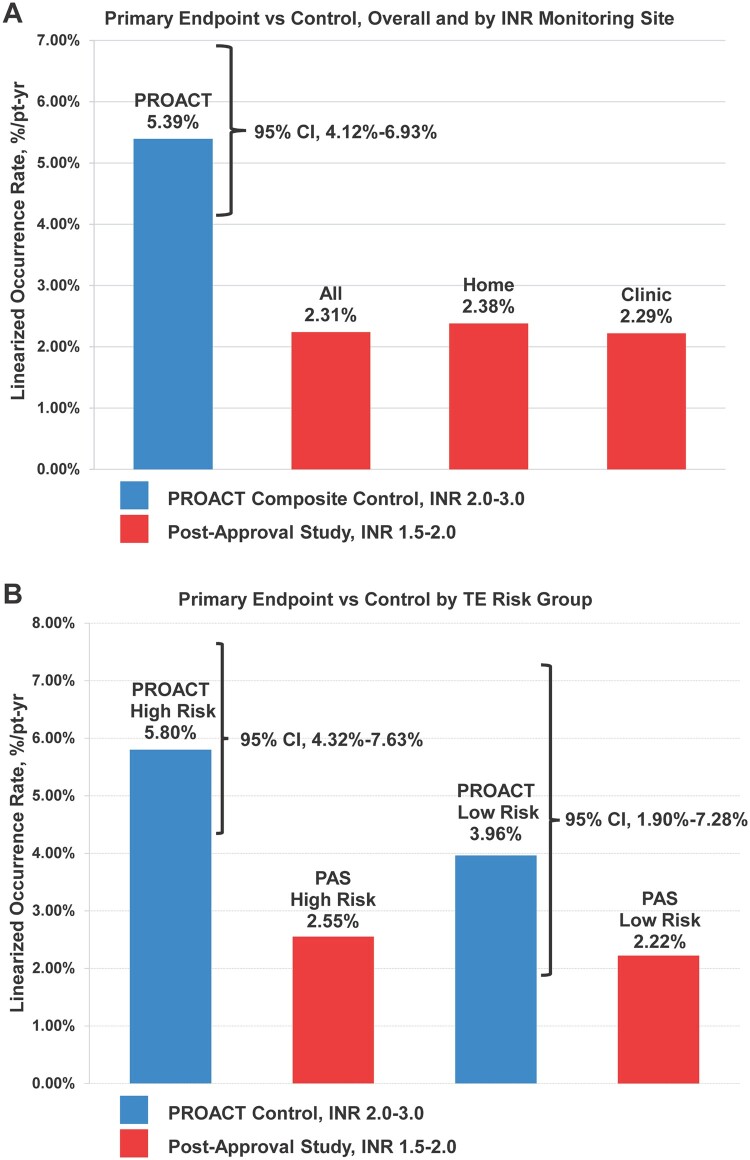
Primary Composite End point versus PROACT Control. (**A**) Primary end point rates overall and by INR monitoring method are displayed next to comparable rate in the PROACT composite control group. (**B**) Primary end point rates in the HR and LR subgroups are displayed next to comparable rates in the HR and LR PROACT control groups. CI: confidence interval; HR: high-risk; INR: international normalized ratio; LR: low-risk; PAS: post-approval study; PROACT: Prospective Randomized On-X Valve Anticoagulation Clinical Trial; pt-yr: patient-year; TE: thromboembolism.

**Figure 3:**
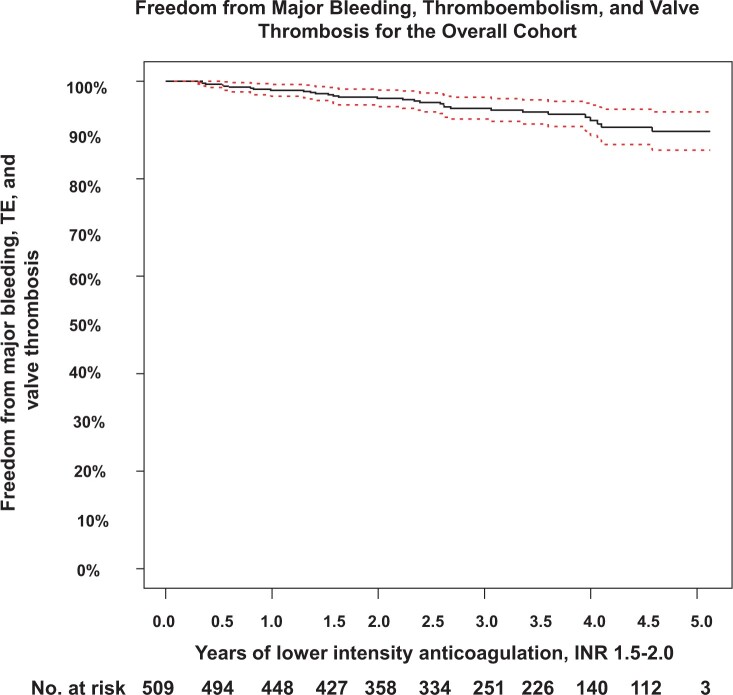
Kaplan–Meier curve for freedom from primary composite end point. In the overall cohort, minimum estimate of freedom from TE, VT or MB is 89.7%. The dotted lines represent the 95% confidence intervals. INR: international normalized ratio; MB: major bleeding; TE: thromboembolism; does not include 1 peripheral TE.

The primary composite end point rate of TE, VT and MB was 2.3% [95% confidence interval (CI) 1.6–3.1%) per pt-yr vs 5.4% (95% CI 4.1–6.9%) in the PROACT composite control group of HR and LR patients on SD warfarin (Table 2 and Fig. 2A). The rate was less than the 95% confidence bound of the control, supporting the study hypothesis, and the incidence rate ratio (IRR) was 0.43. Figure 3 shows the Kaplan–Meier time-to-first-event curve for freedom from TE, VT or MB. The minimum estimated freedom from a primary study end point event was 89.7% at 5 years (standard error 2.0%).

#### Home-monitored and clinic-monitored subgroups

The primary end point rate (%/pt-yr) was 2.4% among home-monitored patients and 2.3% among clinic-monitored patients compared with 5.4% (95% CI 4.1–6.9%) in the PROACT composite control group (IRR 0.44 and 0.43, respectively). Both were better than the 95% CI (Table 3 and Fig. 2A).

#### High-risk and low-risk subgroups

In the HR subgroup, the primary composite end point rate was 2.5% per pt-yr compared with 5.8% in the PROACT HR control group (IRR 0.43) and was better than the 95% CI of 4.3–7.6%. (Table 4, Fig. 2B).

In the LR subgroup, the primary composite end point rate (%/pt-yr) was 2.2% vs 4.0% (95% CI 1.9–7.3%) in the PROACT LR control group (IRR 0.55), and within the 95% CI (Table 4, Fig. 2B).

### Secondary end points

#### Individual components of the primary end point

The rate of MB (0.6%/pt-yr) was markedly less than in the PROACT composite control group (3.8%/pt-yr), with an IRR of 0.15 (Table 2). The 1.7% per pt-yr rate of TE (stroke, TIA and peripheral TE) in the post-approval study was similar to, i.e. within the 95% confidence bound, that of the PROACT composite control group (1.4%/pt-yr, 95% CI 0.81–2.30%). No evidence of VT events (0.0%/pt-yr) has been reported in the post-approval study; the rate in the PROACT composite control group was also low [0.2%/pt-yr (95% CI 0.02–0.6%)]. The rate of TE plus VT overall was 1.7% per pt-yr compared with 1.6% (TE 1.4% plus VT 0.2%) in the PROACT composite control group (IRR 1.09) (Table 2).

#### Patient subsets

Table 5 displays the rates of the composite end point, bleeding and TE in 4 subsets of home- and clinic-monitored HR, and home- and clinic-monitored LR patients. The subsets were not randomized, and statistical comparisons were not performed between subsets. No MBs occurred in the 2 subsets of home-monitored patients.

**Table 5:** Post-approval study event rates by subsets^a^

	Linearized occurrence rate, %/pt-yr (number of events)			
	High risk, home INR	High risk, clinic INR	Low risk, home INR	Low risk, clinic INR
Number of subjects	20	108	50	332
Patient-years^b^	52.6	340.2	157.1	1013.1
Primary end point				
MB, TE, VT	3.80% (2)	2.35% (8)	1.91% (3)	2.27% (23)
Secondary and other end points				
MB	0.00% (0)	0.29% (1)	0.00% (0)	0.79% (8)
TE^c^	3.80% (2)	2.06% (7)	1.91% (3)	1.48% (15)
VT	0.00% (0)	0.00% (0)	0.00% (0)	0.00% (0)
TE and VT	3.80% (2)	2.06% (7)	1.91% (3)	1.48% (15)
Minor bleeding	1.90% (1)	0.88% (3)	1.91% (3)	1.38% (14)
All bleeding	1.90% (1)	1.18% (4)	1.91% (3)	2.17% (22)
Haemorrhagic stroke	0.00% (0)	0.00% (0)	0.00% (0)	0.20% (2)
Ischaemic stroke	3.80% (2)	1.47% (5)	1.91% (3)	0.69% (7)
Non-thrombotic ischaemic stroke	0.00% (0)	0.29% (1)	0.00% (0)	0.10% (1)
TIA	0.00% (0)	0.59% (2)	0.00% (0)	0.69% (7)
Peripheral TE	0.00% (0)	0.00% (0)	0.00% (0)	0.10% (1)
Sudden death	0.00% (0)	0.29% (1)	0.00% (0)	0.10% (1)
Valve-related mortality	0.00% (0)	0.00% (0)	0.00% (0)	0.00% (0)
Total mortality	0.00% (0)	1.47% (5)	0.64% (1)	0.79% (8)

a LORs are calculated based on late patient-years; end points occurring prior to low INR initiation date are excluded.

b Patient-years in late follow-up, i.e. once LD anticoagulation initiated.

c Ischaemic stroke, TIA, and peripheral TE.

INR: international normalized ratio; LOR: linearized occurrence rate; pt-yr(s): patient-year(s); MB: major bleeding; TE: thromboembolism; TIA: transient ischaemic attack; VT: valve thrombosis.

#### Other

No deaths, reoperations or explants were related to a primary end point event; 14 deaths occurred of which 2 were cardiac and 12 were non-cardiac. One autopsy was performed for a death adjudicated as non-cardiac and not valve-related. Valve explant was required in 5 patients, due to major paravalvular leak in 3 and prosthetic valve endocarditis in 2. All explants occurred in clinic-monitored patients.

Two sudden deaths (0.1%/pt-yr overall) occurred. One occurred in the HR, clinic-monitored subset and was adjudicated as sudden cardiac death but not valve-related. The other death occurred in the LR, clinic-monitored subset and was adjudicated as sudden non-cardiac. The sudden cardiac death occurred in a patient found unresponsive at home who was on dialysis for end-stage renal disease.

#### INR <1.5 or >3.0

INR was <1.5 at 6.9% (148 of 2160) of follow-up visits, with 25.9% (66 of 255) of complications, and 8.9% (214 of 2415) of all readings (follow-up visits and complications). INR was >3.0 at 3.9% (85 of 2160) of follow-up visits, with 14.9% (38 of 255) of complications, and 5.1% (123 of 2415) of all readings.

### Tertiary end points

Rates of minor bleeding, all bleeding, haemorrhagic stroke, ischaemic stroke, TIA and peripheral TE are displayed in Tables 2–5. No reoperations, explants or deaths were associated with valve-related TE, VT or MB.

#### Total bleeding

Of note, the rate of total bleeding (%/pt-yr) was 1.9% overall and much lower than the 7.1% (95% CI 5.6–8.8%) rate (IRR 0.27) in the PROACT composite control group of patients on SD warfarin (Table 2).

## DISCUSSION

The clinical registry was initiated following FDA approval to validate the findings of the PROACT trial for the On-X aortic valve. Further, it addresses the question, ‘Is LD warfarin safe for patients with an On-X aortic valve, regardless of INR monitoring method?’ The PROACT trial assessed safety of LD warfarin combined with LD daily aspirin only in patients on home-monitoring at HR of TE. The post-approval study included patients on home or clinic monitoring and high or low risk of TE. Study results will guide patients and clinicians regarding anticoagulation with an On-X aortic valve.

All eligible patients at 23 centres were offered enrolment. Five years of follow-up are planned for each patient, and this interim report includes data through minimum 1 year of follow-up on all patients. The interim analysis shows the rate of the primary composite end point is less than the 95% confidence bound of the comparable rate in the PROACT composite control group, supporting the prespecified study hypothesis. The results are consistent across subgroups of home-monitored, clinic-monitored and HR patients. The individual rates of MB and total bleeding in the overall cohort were much lower than in the PROACT composite control group, with reductions of 85% and 73%, respectively. Rates of TE and VT were similar to those in the PROACT composite control group. In that regard, these results are similar to those of the PROACT trial, demonstrating reduced rates of bleeding with LD (target INR 1.5–2.0) warfarin and daily LD (75–100 mg) aspirin, and similar rates of TE. Our results are also consistent with those reported in 2 randomized controlled trials of LD anticoagulation—LOWERing the INtensity of oral anticoaGulant Therapy (LOWERING-IT) and Early Self-Controlled Anticoagulation Trial III (ESCAT III)—which used wider and higher target INR ranges (1.5–2.5 for LOWERING-IT, 1.6–2.1 for ESCAT III), other mechanical valves and no aspirin [12, 13]. Nonetheless, all 3 studies showed lower rates of bleeding and similar rates of TE with the lower target INR as compared to standard INR management of 2.0–3.0. Only the On-X aortic valve is recommended for this LD INR target (1.5–2.0) in the European and US guidelines.

The value of home INR monitoring has been confirmed in prior studies [14]. We found no MB episodes among home-monitored patients. Only 70 (13.7%) patients were home-monitored, which suggests the existence of perceived or actual barriers to home INR testing. These may be financial, insurance-related, logistical or due to patient or physician preference.

A systematic review of home INR monitoring suggested decreased rates of TE among home-monitored patients, likely due to increased time in therapeutic range [14]. In our study, there were numerically higher rates of TE and ischaemic stroke among home-monitored patients, while rates of TIA were numerically higher in clinic-monitored patients.

The HR group had numerically higher rates of TE and ischaemic stroke. The LR group had numerically higher rates of major, minor and all bleeding.

### Limitations

Limitations of this study include its observational nature and lack of randomization to INR monitoring setting. Patients with differing characteristics may have chosen to enrol or not, resulting in study bias. No method to control for selection bias was used because the study was designed as an all-comer registry to collect real-world data. Not all INR values were captured. INR readings were obtained for an annual visit with the following physician, and those documented when a complication such as a hospitalization occurred. The use of a historical control group might also bias the study. End points were defined only by clinical events, and imaging parameters were not required or included in the analysis. In addition, the analysis did not examine the possible impact of different valve sizes on the end points. Study strengths include its prospective approach, inclusion of patients from multiple centres in 3 countries and enrolment of all willing, eligible patients.

## CONCLUSIONS

Interim results from this post-approval clinical registry of LD warfarin (target INR 1.8, range 1.5–2.0) and daily LD aspirin (75–100 mg) in patients with an On-X aortic valve support the study hypothesis, which states that the rate of the composite primary end point of TE, VT and MB among patients in the post-approval study will be better (less) than the comparable rate in patients on SD warfarin (target INR 2.0–3.0) with daily aspirin (81 mg) in the historical PROACT control group. Results are consistent among subgroups of home-monitored, clinic-monitored and HR patients. The rate of MB was reduced by 85%, and the rate of total bleeding was reduced by 73%, among patients receiving LD warfarin compared to the control on SD warfarin. Rates of TE were similar in the 2 groups. Interim results of this real-world study support the key conclusion from the initial PROACT study that the On-X aortic valve is safe and effective with an INR target range of 1.5–2.0 and LD aspirin, and furthermore suggest that this can be accomplished with or without home INR monitoring.

## Supplementary Material

ezae117_Supplementary_Data

## Data Availability

Dataset is from an ongoing clinical study, which is continuing to collect further follow-up data; not provided.

## Notes

Presented at the 37th Annual Meeting of the European Association of Cardio-Thoracic Surgery, Austria Center Vienna, Vienna, Austria, 5 October 2023.

## ABBREVIATIONS

AE: Adverse event
CI: Confidence interval
ESCAT III: Early Self-Controlled Anticoagulation Trial III
FDA: Food and Drug Administration
HR: High-risk
INR: International normalized ratio
IRR: Incidence rate ratio
LD: Low-dose
LOWERING-IT: LOWERing the INtensity of oral anticoaGulant Therapy
LR: Low-risk
MB: Major bleeding
PROACT: Prospective Randomized On-X Valve Anticoagulation Clinical Trial
pt-yr: Patient-year
SD: Standard-dose
TE: Thromboembolism
TIA: Transient ischaemic attack
VT: Valve thrombosis
