# Supplementary material for: On-X aortic valve replacement patients treated with low-dose warfarin and low-dose aspirin
Source: Eur J Cardiothorac Surg. 2024 Apr 15;65(5):ezae117. doi: 10.1093/ejcts/ezae117 (PMC11136449; doi:10.1093/ejcts/ezae117)
Supplement: ezae117_Supplementary_Data [file ezae117_supplementary_data.docx]

**Supplementary Tables S1-S5, Supplementary Figure S1**

**Supplementary Table S1.** Institutional Review Board Approvals

| **Institution** | **IRB Approval Date** | **IRB Approval ID#** |
| --- | --- | --- |
| Baylor Scott & White  Plano, Texas, US | 27 July 2018 | 018-210 |
| Blackpool Victoria Hospital  Blackpool, Lancashire, England, UK | 8 September 2017 | 17/SC/0191 |
| Carilion Clinic Cardiothoracic Surgery  Roanoke, Virginia, US | 7 August 2018 | 20151431 |
| Cleveland Clinic  Cleveland, Ohio, US | 23 June 2016 | 16-722 |
| Franciscan St. Francis Health  Indianapolis, Indiana, US | 21 April 2016 | 20151431 |
| Hartford Healthcare  Hartford, Connecticut, US | 6 August 2015 | HHC-2015-0186 |
| Hull and East Yorkshire Hospitals  Hull, England, UK | 8 September 2017 | 17/SC/0191 |
| MaineHealth  Portland, Maine, US | 8 December 2016 | 20151431 |
| MultiCare Health  Tacoma, Washington, US | 11 February 2016 | 20151431 |
| Ottawa Heart Institute  Ottawa, Ontario, Canada | 30 October 2018 | 20180501-01H |
| Oxford Heart Centre, John Radcliffe Hospital  Oxford, England, UK | 8 September 2017 | 17/SC/0191 |
| Providence St. Vincent Medical Center  Portland, Oregon, US | 1 August 2018 | STUDY2018000294 |
| Sanford Medical Center  Fargo, North Dakota, US | 3 November 2015 | 20151431 |
| Sentara Norfolk General Hospital  Norfolk, Virginia, US | 8 December 2015 | 20151431 |
| St. Bartholomew’s Hospital  London, England, UK | 8 September 2017 | 17/SC/0191 |
| Southampton University Hospital  Southampton, England, UK | 8 September 2017 | 17/SC/0191 |
| Swedish Med Center  Seattle, Washington, US | 11 October 2015 | 20151431 |
| The Christ Hospital, Lindner Center  Cincinnati, Ohio, US | 21 September 2015 | TCH#15-41 |
| University Hospital Southampton  Southampton, England, UK | 8 September 2017 | 17/SC/0191 |
| University of Calgary Foothills  Calgary, Albert, Canada | 4 May 2018 | REB18-0458 |
| UT Health Science Center at Houston  Houston, Texas, US | 2 December 2016 | HSC-MS-16-0471 |
| UT Southwestern  Dallas, Texas, US | 7 November 2016 | STU 092015-071 |
| Victoria Heart Institute  Victoria, British Columbia, Canada | 1 August 2018 | C2018-036 |

**Supplementary Table S2.** On-X Valve Models and Sizes in Post-Approval Study

| **Device Trade Name** | **Model Designator** | |
| --- | --- | --- |
|  | With Standard Holder | With Extended Holder |
| On-X^®^ Prosthetic Heart Valve (Aortic) | ONXA-19  ONXA-21  ONXA-23  ONXA-25  ONXA-27/29 | ONXAE-19  ONXAE-21  ONXAE-23  ONXAE-25  ONXAE-27/29 |
| On-X^®^ Conform-X^®^ Aortic  Prosthetic Heart Valve | ONXAC-19  ONXAC-21  ONXAC-23  ONXAC-25  ONXAC-27/29 | ONXACE-19  ONXACE-21  ONXACE-23  ONXACE-25  ONXACE-27/29 |
| On-X^®^ Aortic Prosthetic  Heart Valve with Anatomic  Sewing Ring | ONXAN-19  ONXAN-21  ONXAN-23  ONXAN-25  ONXAN-27/29 | ONXANE-19  ONXANE-21  ONXANE-23  ONXANE-25  ONXANE-27/29 |

**Supplementary Table S3.** Historical Control Rates from PROACT Trial

|  | **High-Risk Control^a^**  **878.6 pt-yrs** | | **Low-Risk Control^b^**  **252.6 pt-yrs** | | **Composite Control^c^**  **1131.2 pt-yrs** | |
| --- | --- | --- | --- | --- | --- | --- |
| **Adverse Event** | LOR  (%/pt-yr) | 95% CI | LOR  (%/pt-yr) | 95% CI | LOR  (%pt-yr) | 95% CI |
| Composite^d^ | 5.80 | 4.32-7.63 | 3.96 | 1.90-7.28 | 5.39 | 4.12-6.93 |
| Major bleeding | 3.87 | 2.68-5.41 | 3.56 | 1.63-6.76 | 3.80 | 2.75-5.12 |
| Thromboembolism^e^ | 1.70 | 0.96-2.82 | 0.40 | 0.01-2.21 | 1.41 | 0.81-2.30 |
| Valve thrombosis | 0.23 | 0.03-0.82 | 0.00 | 0.0-1.46 | 0.18 | 0.02-0.64 |
| Total bleeding | 7.85 | 6.11-9.94 | 4.53 | 2.17-7.79 | 7.07 | 5.61-8.80 |

Abbreviations: CI, confidence interval from the Poisson distribution; LOR, linearized occurrence rate; pt-yr(s), patient-year; TE, thromboembolism.

^a^ From P000037/S030. ^b^ From G050208 2014 Annual Report. ^c^ Weighted average of control groups.

^d^ Major bleeding, total TE, valve thrombosis. ^e^ Ischemic stroke, TIA, and peripheral TE.

**Supplementary Table S4**. Adverse Event Definitions

The Post-Approval Clinical Registry Study utilizes adverse event definitions which were established in guidelines issued in 2008 by the American Association for Thoracic Surgery, The Society of Thoracic Surgeons, and the European Association for

Cardio-Thoracic Surgery. These definitions are stated below.

Reference:

Akins CW, Miller DC, Turina MI, Kouchoukos NT, Blackstone EH, Grunkemeier GL, *et al.* Guidelines for reporting mortality and morbidity after cardiac valve interventions; J Thorac Cardiovasc Surg 2008; 135:732-8.

Bleeding Event

*A bleeding* *event* is any episode of major internal or external bleeding that causes death, hospitalization, or permanent injury (e.g., vision loss) or necessitates transfusion. Major bleeding unexpectedly associated with minor trauma should be reported as a *bleeding event*, but bleeding associated with major trauma or a major operation should not. *Bleeding events* are reported for all patients regardless of whether they are taking anticoagulants or antiplatelet drugs. Although total *bleeding events* must be reported, *bleeding events* can also be reported separately for those who are taking anticoagulants or antiplatelet agents and those who are not.

Embolism

*Embolism* is any embolic event that occurs in the absence of infection after the immediate perioperative period. *Embolism* may be manifested by a *neurologic event or a noncerebral embolic event.*

A *neurologic event* includes any central, new neurologic deficit, whether temporary or permanent and whether focal or global, that occurs after the patient emerges from anesthesia.

*Stroke* is a prolonged (> 72 hours) or permanent neurologic deficit that is usually associated with abnormal results of magnetic resonance imaging or computed tomographic scans. Patients with minimal, atypical, or protean symptoms that lead to radiographic imaging demonstrating an acute ischemic event are considered to have sustained a *stroke*.

*Transient ischemic attack* is characterized by fully reversible symptoms of short duration. If radiographic imaging demonstrates an acute central neurologic lesion (“cerebral infarction with transient symptoms"), however, such patients are reclassified as having sustained a *stroke*.

Multiple or repeated transient events occurring during a short period (a burst or *cluster*) should be recorded as one event for calculation of event rates, but documented as a *cluster*. Rate calculations should be provided not only for all embolic events but also separately for *strokes, transient ischemic attacks*, and *clusters*.

Postoperative neurologic symptoms that mimic those of a preoperatively documented neurologic event and that are confirmed radiographically to be consistent with the former event are not counted as a new neurologic event. Central neurologic events that are clearly related to aortic, internal carotid artery, or vertebral artery disease, such as acute thrombotic occlusion, atheroembolism, or spontaneous arterial dissection, are also not counted.

*Psychomotor deficits* found by specialized testing are not considered neurologic events related to operated valves. Patients who do not awaken or who awaken after operation with a new stroke are not considered to have sustained valve-related neurologic events.

A *noncerebral embolic* event is an embolus documented operatively, at autopsy, or clinically that produces signs or symptoms attributable to complete or partial obstruction of a peripheral artery. Intraoperative myocardial infarctions are not counted. Postoperative myocardial infarction is also not counted unless the infarction is caused by a coronary embolus (as detected by operation, autopsy, or clinical imaging). Emboli consisting of non-thrombotic material (e.g., atherosclerosis, myxoma) are not counted.

Valve Thrombosis

*Valve thrombosis* is any thrombus not caused by infection attached to or near an operated valve that occludes part of the blood flow path, interferes with valve function, or is sufficiently large to warrant treatment. Valve thrombus found at autopsy in a patient whose cause of death was not valve-related or found at operation for an unrelated indication should also be counted as *valve thrombosis*.”

Reintervention

*Reintervention* is any surgical or percutaneous interventional catheter procedure that repairs, otherwise alters or adjusts, or replaces a previously implanted prosthesis or repaired valve. In addition to surgical reoperations, enzymatic, balloon dilatation, interventional manipulation, repositioning, or retrieval, and other catheter-based interventions for valve-related complications are also considered *reinterventions*. Indications for *reintervention* must be reported. Open surgical and percutaneous catheter *reinterventions* should be listed separately.

Valve-Related Mortality

*Valve-related mortality* is any death caused by *structural valve deterioration*, *nonstructural dysfunction*, valve *thrombosis*, *embolism*, *bleeding event*, or *operated valve endocarditis*; death related to *reintervention* on the operated valve; or *sudden*, *unexplained death*. Deaths caused by heart failure in patients with advanced myocardial disease and satisfactorily functioning cardiac valves are not counted. Specific causes of valve-related deaths should be reported.

Sudden, Unexplained Death

A *sudden, unexplained death* is one in which the cause of death has not been determined by clinical investigation or autopsy findings and the relationship to the operated valve is undefined. These deaths should be reported as a separate category, but also included in *valve-related mortality*.

**Supplementary Table S5.** Patients Discontinued From the Post-Approval Study

| **Subgroups** | **High-Risk**  **n = 128** | **Low-Risk**  **n = 382** | **Clinic**  **n= 440** | **Home**  **n = 70** | **All**  **N = 510** | |
| --- | --- | --- | --- | --- | --- | --- |
| Deaths | 5 (3.9%) | 9 (2.4%) | 13 (3.0%) | 1 (1.4%) | 14 (2.7%) | |
| Explants | 2 (1.6%) | 3 (0.8%) | 5 (1.1%) | 0 (0.0%) | 5 (1.0%) | |
| LTF | 2 (1.6%) | 7 (1.8%) | 8 (1.8%) | 1 (1.4%) | 9 (1.8%) | |
| Withdrawn^a^ | 6 (4.7%) | 8 (2.1%) | 7 (1.6%) | 7 (10.0%) | 14 (2.7%) | |
| Other^b^ | 3 (2.3%) | 6 (1.6%) | 8 (1.8%) | 1 (1.4%) | 9 (1.8%) | |
| Total | 18 (14.1%) | 33 (8.6%) | 41 (9.3%) | 10 (14.3%) | 51 (10.0%) | |
| **Subsets** | **High-Risk**  **Clinic**  **n = 108** | **High-Risk**  **Home**  **n = 20** | **Low-Risk**  **Clinic**  **n = 332** | **Low-Risk**  **Home**  **n = 50** | **All**  **N = 510** |  |
| Deaths | 5 (4.6%) | 0 (0.0%) | 8 (2.4%) | 1 (2.0%) | 14 (2.7%) |  |
| Explants | 2 (1.9%) | 0 (0.0%) | 3 (0.9%) | 0 (0.0%) | 5 (1.0%) |  |
| LTF | 1 (0.9%) | 1 (5.0%) | 7 (2.1%) | 0 (0.0%) | 9 (1.8%) |  |
| Withdrawn^a^ | 3 (2.8%) | 3 (15.0%) | 4 (1.2%) | 4 (8.0%) | 14 (2.7%) |  |
| Other^b^ | 2 (1.9%) | 1 (5.0%) | 6 (1.8%) | 0 (0.0%) | 9 (1.8%) |  |
| Total | 13 (12.0%) | 5 (25.0%) | 28 (8.4%) | 5 (10.0%) | 51 (10.0%) |  |

Abbreviations: LTF, lost to follow-up.

^a^ Withdrawn due to patient or investigator decision. ^b^ Discontinued for another reason.

**Supplementary Figure S1.** Patients Discontinued by Subgroup and Subset


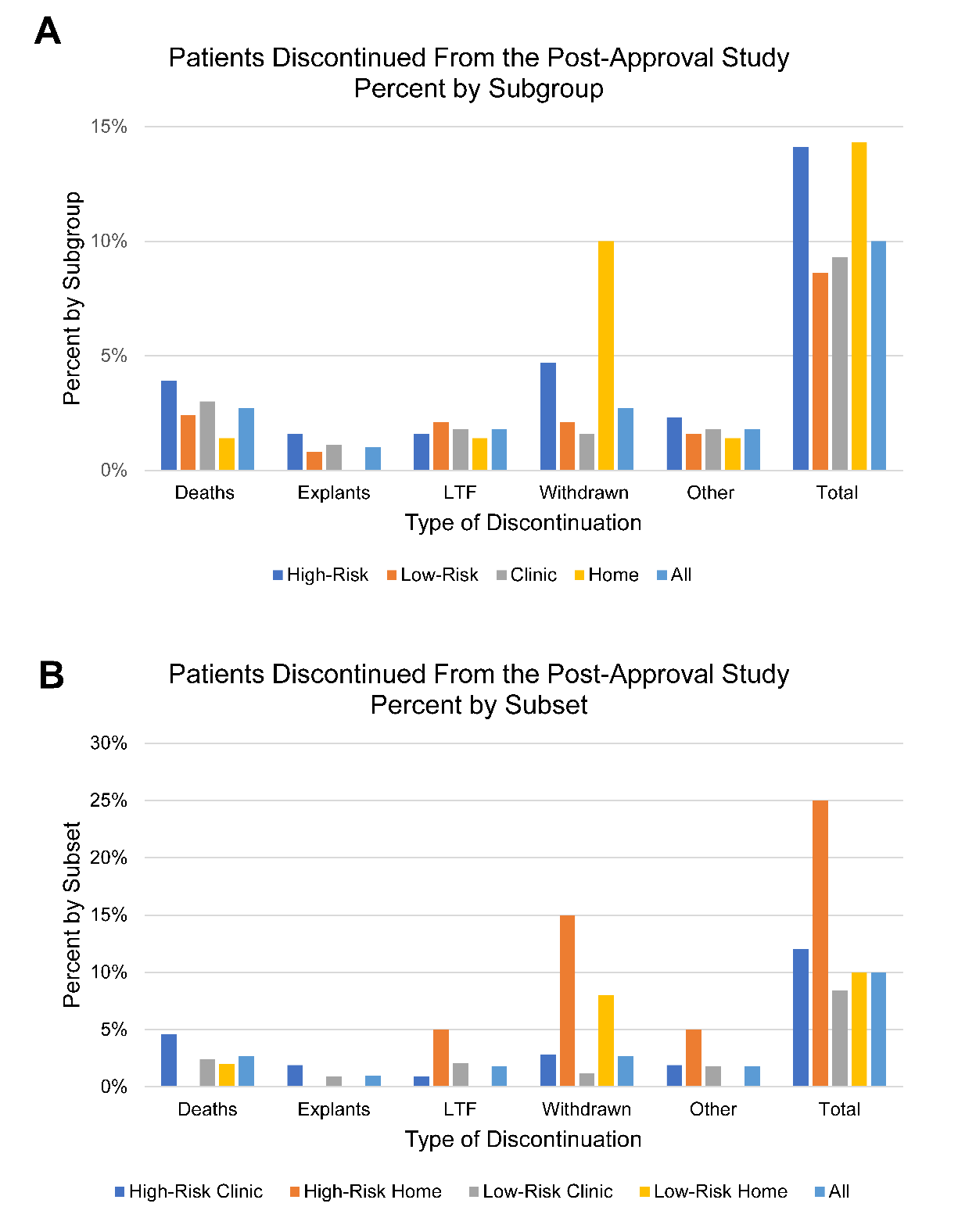


**Supplementary Figure 1*.*** Patients Discontinued by Subgroup and Subset.

**(A)** Patients Discontinued by Subgroup. **(B)** Patients Discontinued by Subset.

These figures show there were more deaths and explants in the high-risk group and clinic-monitored groups than in the low-risk and home-monitored groups. Patients lost to follow-up were relatively evenly distributed across the subgroups and subsets. A greater percentage of high-risk and home-monitored patients were withdrawn due to patient or investigator decision. The absolute number of withdrawals, however, was very similar (6 high-risk, 8 low-risk, 7 clinic, 7 home.)
